# Differential Interventional Effects of Omega-6 and Omega-3 Polyunsaturated Fatty Acids on High Fat Diet-Induced Obesity and Hepatic Pathology

**DOI:** 10.3390/ijms242417261

**Published:** 2023-12-08

**Authors:** Lei Hao, Chih-Yu Chen, Yong-Hui Nie, Kanakaraju Kaliannan, Jing X. Kang

**Affiliations:** 1 Laboratory for Lipid Medicine and Technology (LLMT), Department of Medicine, Massachusetts General Hospital and Harvard Medical School, Boston, MA 02129, USA; leihao@iup.edu (L.H.); chih-yu.chen@emory.edu (C.-Y.C.);; 2 Department of Nursing and Allied Health Professions, Indiana University of Pennsylvania, Indiana, PA 15705, USA; 3 Emory School of Medicine, Emory University, Atlanta, GA 30322, USA; 4 Omega-3 and Global Health Institute, Boston, MA 02129, USA

**Keywords:** obesity, omega-3 fatty acids, omega-6 fatty acids, n-6/n-3 fatty acid ratio, non-alcoholic fatty liver disease, lipogenesis

## Abstract

Current Dietary Guidelines for Americans recommend replacing saturated fat (SFA) intake with polyunsaturated fatty acids (PUFAs) and monosaturated fatty acids (MUFAs) but do not specify the type of PUFAs, which consist of two functionally distinct classes: omega-6 (n-6) and omega-3 (n-3) PUFAs. Given that modern Western diets are already rich in n-6 PUFAs and the risk of chronic disease remains high today, we hypothesized that increased intake of n-3 PUFAs, rather than n-6 PUFAs, would be a beneficial intervention against obesity and related liver diseases caused by high-fat diets. To test this hypothesis, we fed C57BL/6J mice with a high-fat diet (HF) for 10 weeks to induce obesity, then divided the obese mice into three groups and continued feeding for another 10 weeks with one of the following three diets: HF, HF+n-6 (substituted half of SFA with n-6 PUFAs), and HF+n-3 (substituted half of SFA with n-3 PUFAs), followed by assessment of body weight, fat mass, insulin sensitivity, hepatic pathology, and lipogenesis. Interestingly, we found that the HF+n-6 group, like the HF group, had a continuous increase in body weight and fat mass, while the HF+n-3 group had a significant decrease in body weight and fat mass, although all groups had the same calorie intake. Accordingly, insulin resistance and fatty liver pathology (steatosis and fat levels) were evident in the HF+n-6 and HF groups but barely seen in the HF+n-3 group. Furthermore, the expression of lipogenesis-related genes in the liver was upregulated in the HF+n-6 group but downregulated in the HF+n-3 group. Our findings demonstrate that n-6 PUFAs and n-3 PUFAs have differential effects on obesity and fatty liver disease and highlight the importance of increasing n-3 PUFAs and reducing n-6 PUFAs (balancing the n-6/n-3 ratio) in clinical interventions and dietary guidelines for the management of obesity and related diseases.

## 1. Introduction

Fat is a macronutrient that provides energy and regulates many biological pathways in the human body [1]. Fatty acids can be classified into saturated fatty acids (SFAs), monosaturated fatty acids (MUFAs), and polyunsaturated fatty acids (PUFAs). PUFAs, including omega-6 (n-6) and omega-3 (n-3), are essential nutrients and key modulators of diverse biological processes and cell membrane properties [2,3]. Currently, the 2020 Dietary Guidelines for Americans recommend limiting SFAs intake to less than 10% total energy intake and replacing SFAs with PUFAs and MUFAs [4]. However, these guidelines do not clearly state what types of PUFAs should be consumed and the quantities of PUFAs that should be consumed.

Omega-6 (n-6) and omega-3 (n-3) PUFAs exhibit distinct metabolic and functional characteristics. They compete for the same enzymes for biosynthesis and metabolism and have opposing effects on many physiological and pathological processes—including inflammation, gut microbiota, energy metabolism, insulin sensitivity, and lipogenesis—which underlie the development of many chronic diseases [5,6,7,8,9,10]. For example, n-6 PUFAs generally promote inflammation, whereas n-3 PUFAs have anti-inflammatory properties through multiple mechanisms [11,12,13].

Contemporary farming practices, marked by a reliance on grain-based diets, have contributed to a rise in overall SFAs and the presence of n-6 PUFAs, such as linoleic and arachidonic acids. These n-6 PUFAs are notably abundant in vegetable oils like corn oil, sunflower seed oil, soybean oil, and safflower oil, and in livestock raised on grain-based diets. Over the last century, factors such as the industrial revolution, the ascendancy of agribusiness focused on processed foods, the feeding of livestock with grains, and the hydrogenation of vegetable fats have collectively diminished the levels of n-3 PUFAs while concurrently elevating the levels of n-6 PUFAs [5,14,15]. In the United States, the consumption of n-6 linoleic acid has more than doubled over the past century [5]. Consequently, modern diets in many countries are deficient in n-3 PUFAs and have too many n-6 PUFAs, resulting in a high n-6/n-3 PUFA ratio. According to some estimates, the typical Western diet has an n-6/n-3 ratio ranging from 10:1 to 20:1, which is much higher than the recommended ratio of 4:1 or lower for optimal health [5].

The increase in the n-6/n-3 PUFAs ratio corresponds with a notable rise in the prevalence of conditions such as overweight, obesity, diabetes, and cancer [16]. Numerous observational studies have demonstrated a link between higher n-6/n-3 ratios and an elevated risk of obesity and metabolic syndrome [16]. Consistent findings from various animal studies, including those involving trials with n-3 PUFA supplements and the transgenic fat-1 mouse model, suggest that n-3 PUFAs have preventive effects against obesity and other metabolic disorders [12,16]. In addition, some studies have assessed the efficacy of incorporating n-3 PUFAs into a high-fat diet (HF) midway through the study to reverse diet-induced weight gain and associated metabolic alterations [12]. However, the results from these interventional studies are varied, with some not showing that n-3 PUFAs can reverse obesity [12,17,18]. Taken together, it is important to note that prior research primarily focused on the preventive effects of n-3 PUFAs on obesity, often administering supplementation concurrently with the initiation of an HF diet. Nevertheless, the therapeutic potential of both n-3 and n-6 PUFAs to reverse existing obesity and non-alcoholic fatty liver disease (NAFLD) remains to be investigated.

Our hypothesis posits that supplementing mice with obesity and NAFLD with n-3 PUFAs can lead to a substantial improvement or reversal of their health conditions, whereas supplementation with n-6 PUFAs may have no positive effects or could potentially result in adverse outcomes. This study aims to investigate and delineate the distinct impacts of n-6 and n-3 PUFAs on obesity and NAFLD, offering crucial insights for clinical interventions and the formulation of dietary guidelines.

## 2. Results

### 2.1. The Differential Effects of a High-Fat Diet Rich in n-6 PUFAs or n-3 PUFAs on Obesity

**Figure 1:**
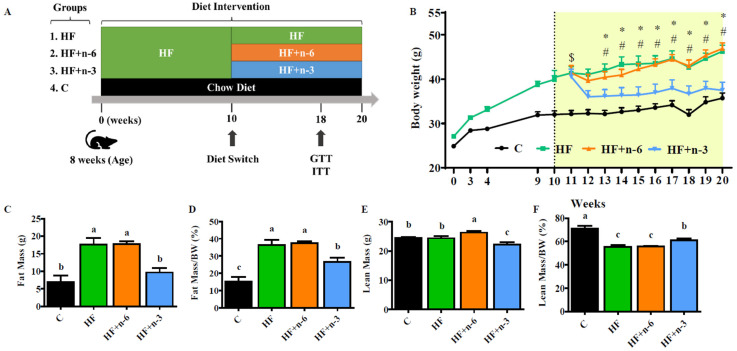
The differential effects of a high-fat diet rich in n-6 PUFAs or n-3 PUFAs on obesity. To investigate the interventional effect of different dietary PUFAs on obesity, male C57BL/6J mice were first fed a high-fat diet (45% kcal from fat, HF) for 10 weeks, and then divided into three groups and continued feeding for another 10 weeks with one of the following three diets: HF, HF+n-6 (substituting half of SFA with n-6 PUFAs), and HF+n-3 (substituting half of SFA with n-3 PUFAs). One group of mice, as controls, were continually fed the HF for another 10 weeks. (**A**) The experimental designs. (**B**) Body weight of mice before and after 10 weeks of intervention. (**C**) Total fat mass. (**D**) Relative fat mass to body weight. (**E**) Total lean mass. (**F**) Relative lean mass to body weight. Data shown are the mean or mean ± S.E.M (*n* = 6–7/group). * *p* < 0.05 HF+n-3 compared to HF, # *p* < 0.05 HF+n-3 compared to HF+n-6, $ *p* < 0.05 All high-fat diet feed groups compared to C, the different letter indicates significant difference.

To investigate the interventional effect of different dietary PUFAs on obesity, male C57BL/6J mice were first fed a high-fat diet (45% kcal from fat, HF) for 10 weeks, and then divided into three groups and continued feeding for another 10 weeks with one of the following three high-fat diets (Figure 1A): HF, HF+n-6 (substituting half of SFA with n-6 PUFAs), and HF+n-3 (substituting half of SFA with n-3 PUFAs) (Supplementary Tables S1 and S2). The calories from protein (20% kcal), carbohydrates (35% kcal), and fat (45% kcal) were designed to be equal in each diet. The n-6/n-3 PUFA ratios of these three diets were 10:1, 20:1, and 0.3:1 in HF, HF+n-6, and HF+n-3, respectively (Supplementary Tables S1 and S2). After 10 weeks of dietary intervention, mice fed HF+n-6 and HF continued to gain weight, while mice fed HF+n-3 showed significant weight loss (Figure 1B). We noticed HF+n-3 mice had dramatic weight loss in week 12, but the cumulative food intake did not exhibit a significant difference between HF and HF+n-3 groups. Consistently, fat mass (total and BW normalized) in the HF+n-3 group was significantly reduced relative to that of the HF and HF+n-6 groups (Figure 1C,D). Although the total lean mass seemed to be higher in the HF+n-6 group (Figure 1E), after adjusting their corresponding body weight, HF+n-3 mice had more lean mass than the mice in the HF and HF+n-6 groups (Figure 1F). Noticeably, although the HF+n-6 and HF+n-3 diets contained the same number of calories and the same level of PUFAs, they exhibited opposite effects on body weight gain after a 10-week dietary intervention (i.e., HF+n-6 with a high n-6/n-3 ratio increased body weight gain whereas HF+n-3 with a low n-6/n-3 ratio reduced body weight).

### 2.2. The Differential Effects of High-Fat Diet Rich in n-6 PUFAs or n-3 PUFAs on Glucose Homeostasis

**Figure 2:**
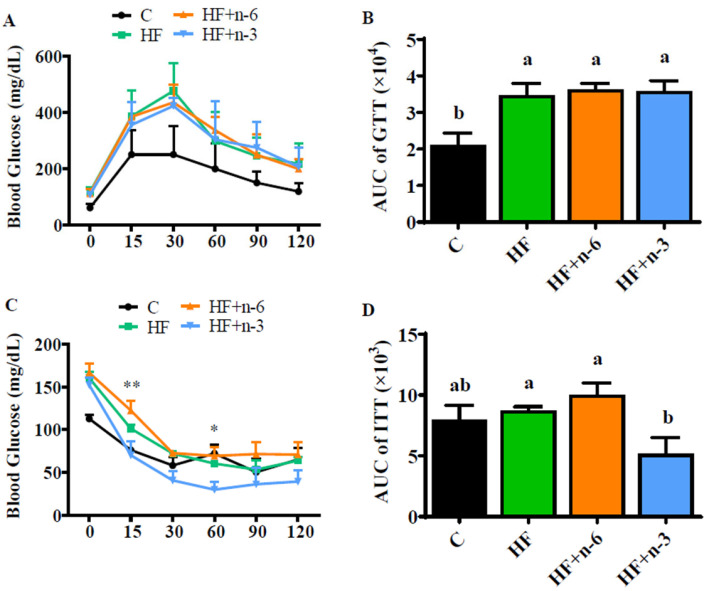
The differential effects of a high-fat diet rich in n-6 PUFAs or n-3 PUFAs on glucose homeostasis. The glucose tolerance test (GTT) and insulin tolerance test (ITT) were conducted after the mice had been switched to intervention diets for 8 weeks. (**A**) The levels of glucose at different time points during GTT. (**B**) The area under the curve is calculated according to the plot of GTT. (**C**) The levels of glucose at different time points during ITT. (**D**) The area under the curve is calculated according to the plot of ITT. Values represent mean ± S.E.M. Error bars represent S.E.M., and different letters indicate significant difference. * *p* < 0.05, ** *p* < 0.01, represents significant difference between HF+n-3 and HF.

To understand whether glucose metabolism is altered by the dietary PUFA interventions, we performed a glucose tolerance test (GTT) and insulin tolerance test (ITT) when the mice had been switched to the intervention diets for 8 weeks. All groups fed with high fat (HF, HF+n-6, HF+n-3) had hyperglycemia characterized by impaired glucose tolerance compared to the C group (Figure 2A,B). The responses to glucose load at each time point and area under curve (AUC) which was calculated based on the plot of GTT were similar among these HF groups (Figure 2A,B). However, the fasting blood glucose level was lower in the HF+n-3 group (104.4 ± 4.2 mg/dL) compared to HF- (117.0 ± 6.5 mg/dL) and HF+n-6 (113.8 ± 6.2 mg/dL)-fed mice, but the difference did not reach statistical significance. Interestingly, ITT results showed that the HF+n-3 group had a much more sensitive response to insulin action compared to the HF+n-6 and HF groups, especially at 15 min and 60 min following insulin administration (Figure 2C). The AUC calculated based on the plot of ITT was also significantly less in the HF+n-3 group than in the HF+n-6 and HF groups (Figure 2D). In summary, although the HF+n-3 intervention did not improve hyperglycemia, it could significantly alleviate HF-induced insulin resistance, while the HF+n-6 diet did not have such a beneficial effect on insulin sensitivity.

### 2.3. The Differential Effects of a High-Fat Diet Rich in n-6 PUFAs or n-3 PUFAs on the Hepatic Pathology

**Figure 3:**
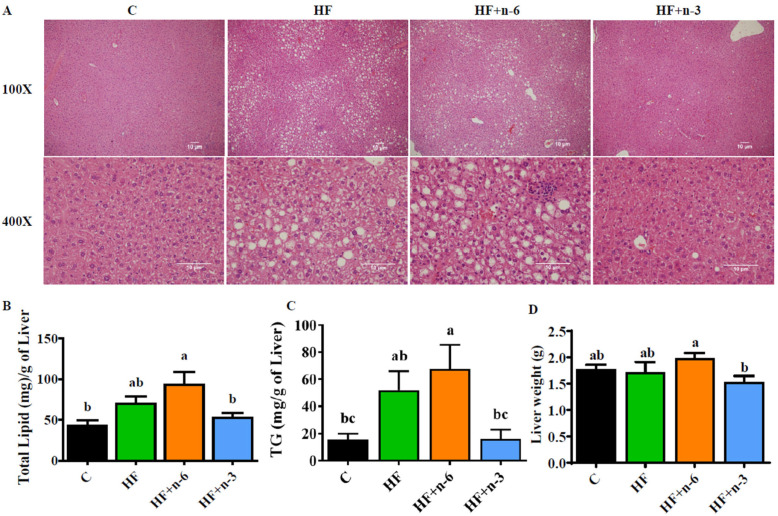
The differential effects of a high-fat diet rich in n-6 PUFAs or n-3 PUFAs on the hepatic pathology. To explore the impacts of diets with different PUFAs on the development of non-alcoholic fatty liver, the histological changes and lipid content in the livers of mice were examined. (**A**) Hematoxylin and eosin (H&E) staining of liver sections. Scale bar = 10 μm. (**B**) Total lipid content per milligram of liver tissue. (**C**) Total TG per milligram of liver tissue. (**D**) Liver weight. Values represent mean ± S.E.M. Error bars represent S.E.M., and different letters indicate significant difference. A significant difference was defined as *p* < 0.05.

After 10 weeks of dietary interventions, the hepatic fatty acid profile exhibited marked difference among the four diet groups with an n-6/n-3 ratio of 2.2, 4.5, 7.5, and 0.6 for C, HF, HF+n-6, and HF+n-3, respectively (Supplementary Table S4). To explore the impacts of the diets with different PUFAs on the development of NAFLD, we examined the histological changes and lipid content in the livers of the animals treated with the diets. Histological examination showed focal inflammation, focal necrosis, and lipid accumulation in hepatocytes from the mice fed with HF and HF+n-6, compared to the control mice on a low-fat diet (Figure 3A). Strikingly, these pathological changes were almost totally reversed by the n-3 PUFA intervention (HF+n-3) (Figure 3A). Analysis of hepatic lipid content showed that the HF+n-3 group exhibited lower levels of total lipid and TG in the liver than the HF+n-6 and HF groups (Figure 3B,C). In addition, the HF+n-3 group had signific lower liver weight compared with the HF+n-6 group (Figure 3D). Collectively, the n-3 PUFA intervention (low dietary ratio of n-6/n-3 PUFAs) could restore HF-induced excess lipid accumulation and inflammation in the liver.

### 2.4. The Differential Effects of High-Fat Diet Rich in n-6 PUFAs or n-3 PUFAs on mRNA Levels of Lipogenic Enzymes in the Liver

**Figure 4:**
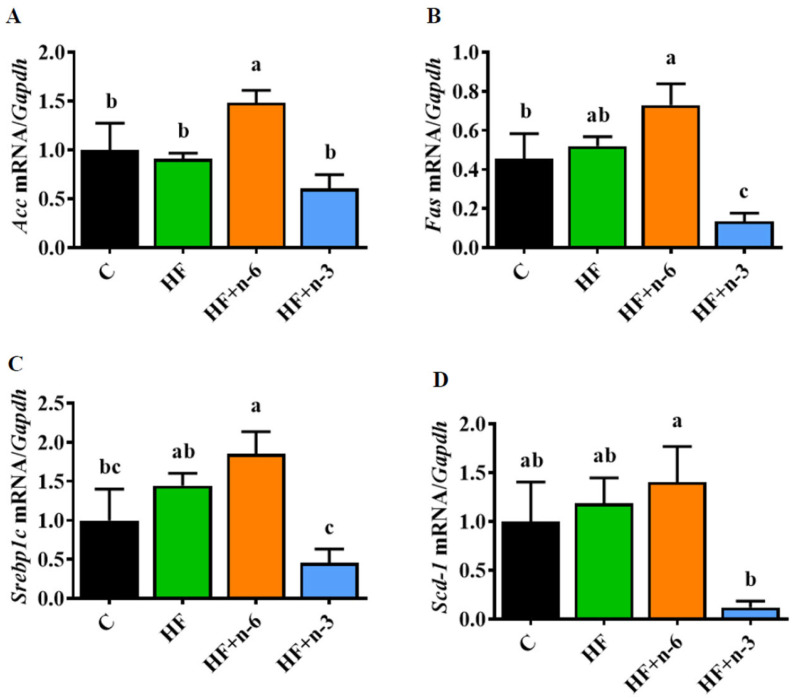
The differential effects of a high-fat diet rich in n-6 PUFAs or n-3 PUFAs on mRNA levels of lipogenic enzymes in the liver. (**A**) Gene expression of *Acc*. (**B**) Gene expression of *Fas*. (**C**) Gene expression of *Srebp1c*. (**D**) Gene expression of *Scd-1*. Error bars represent S.E.M., and different letters indicate significant differences. A significant difference was defined as *p* < 0.05.

As increased de novo lipogenesis is a major contributor to obesity and excessive lipid accumulation in the liver (a prominent abnormality in NAFLD) [19], we next analyzed several molecular targets related to de novo lipogenesis. In general, ATP citrate lyase (ATP-CL) bridges glucose metabolism and fatty acid biosynthesis by generating acetyl-CoA, and sequentially, acetyl-CoA is metabolized to malonyl-CoA as well as fatty acids such palmitate (C16:0) through acetyl-CoA carboxylase (*Acc*), fatty acid synthase (*Fas*), and stearoyl-CoA desaturase 1 (*Scd-1*). These genes are transcriptionally activated by sterol regulatory element-binding protein 1c (*Srebp1c*). In the present study, we found that hepatic mRNA levels of *Acc*, *Fas*, *Scd-1*, and *Srebp1c* were upregulated in mice fed with the HF+n-6 diet but downregulated in the HF+n-3 group (Figure 4A–D).

## 3. Discussion

Obesity is a major public health issue that has become increasingly prevalent worldwide [20]. According to the World Health Organization, more than 650 million adults worldwide were obese in 2016, and the prevalence of obesity has tripled since 1975 [21]. Obesity is a complex condition that involves an imbalance between energy intake and expenditure, leading to an excess accumulation of adipose tissue. This excess adipose tissue can have a negative impact on various physiological systems, including the cardiovascular, respiratory, and endocrine systems [22]. Moreover, obesity is associated with an increased risk of developing several chronic diseases, including type 2 diabetes, cardiovascular disease, and NAFLD [23,24]. NAFLD, which is tightly linked to obesity, is on the rise in incidence across the United States and is currently estimated to affect 25% of the American population, with annual direct medical costs of approximately $103 billion [25]. Current treatment options for obesity and NAFLD include lifestyle modifications, such as diet and exercise, and pharmacological interventions, but these approaches often have limited effectiveness and can be associated with side effects [26,27]. Omega-3 PUFAs have been proposed as a potential therapeutic option due to their anti-inflammatory and lipid-lowering effects [28,29,30].

The present study was designed to address whether n-3 PUFAs or n-6 PUFAs supplementation has an interventional effect on obesity and associated fatty liver. Our results showed that after 10 weeks of dietary interventions (shift of HF to HF+n-3 or HF+n-6), the HF+n-3 diet could significantly reduce body weight and fat mass of obese mice previously maintained on HF whereas the HF+n-6 diet failed to do so, rather, it further increased body weight of the obese mice. These findings support previous studies reporting a beneficial effect of n-3 PUFAs on body weight and adiposity [31,32,33,34,35,36,37,38]. Moreover, the HF+n-3 diet also led to a significant reduction in liver weight and hepatic lipid accumulation compared to the HF and HF+n-6 diets. Thus, our study demonstrates an effective interventional role for n-3 PUFAs in the management of obesity and associated fatty liver and clarifies the differential biological effects of n-6 PUFAs and n-3 PUFAs. On this basis, we recommend that any dietary guidelines or health policies regarding PUFAs should specify the type of PUFA (n-6 or n-3) to ensure their benefits and avoid adverse effects.

Interestingly, our findings also suggest that the n-6/n-3 PUFA ratio may play a role in the development of obesity and NAFLD. Both HF+n-6 and HF+n-3 diets had the same calorie, total PUFA level, and fat content, but different n-6/n-3 PUFA ratios (20:1 and 0.3:1, respectively). The striking difference between HF+n-6 (with a high n-6/n-3 ratio) and HF+n-3 (with a low n-6/n-3 ratio) in their effects on body weight and liver fat content supports the notion that the n-6/n-3 PUFA ratio plays a role in the development of metabolic disorders [7,16].

Most previous studies primarily focused on the preventive effects of n-3 PUFAs on obesity, where supplementation occurred concurrently with the initiation of a high-fat (HF) diet. In contrast, our study was designed to assess the therapeutic effectiveness of n-3 PUFAs by examining their impact on the reversal of obesity. This represents a unique angle in the field. We are aware of studies, such as the one by Huang et al., that explored therapeutic effects [18], but our approach differs in key aspects. For instance, while Huang’s study used a 60% fat diet and had a 6-week intervention period, we employed a 45% fat diet and extended our intervention to 10 weeks. Moreover, our focus on liver lipogenic gene expression distinguishes our work from studies concentrating on brain neuropeptide gene expression.

A significant innovation in our study lies in the simultaneous examination of the therapeutic effects of n-6 and n-3 PUFAs within the same experiment to address their differential effects. To the best of our knowledge, this comprehensive approach has not been previously reported. Furthermore, the use of a 45% high-fat diet in our obese model aligns more closely with human physiology, enhancing the translational relevance of our findings.

**Figure 5:**
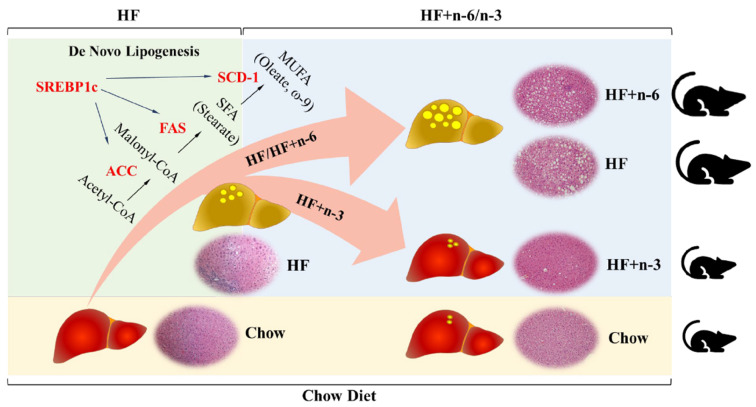
Proposed model of the differential effect of a high-fat diet rich in n-6 PUFAs or n-3 PUFAs on fatty liver and obesity. Schematic model showing proposed mechanism underlying how high-fat diets with different n-6/n-3 ratios differently regulate the development of non-alcoholic fatty liver disease and obesity. SREBP1c: sterol regulatory element-binding transcription factor 1, SCD-1: stearoyl-CoA desaturase 1, FAS: fatty acid synthase, and ACC: acetyl-CoA carboxylase.

One of the potential mechanisms by which n-3 PUFAs exert their beneficial effects observed in this study is by modulating de novo lipogenesis, as the HF+n-3 diet could significantly reduce the abundance of transcripts of genes involved in de novo lipogenesis, which is a key determinant of excessive fat accumulation in adipose tissues and the liver (Figure 4 and Figure 5). One limitation of the study is that we solely focused on gene expression analysis. Future investigations are warranted to delve into how different diets impact the protein levels of these lipogenic enzymes. Certainly, many other mechanisms may be involved as shown previously by various studies [12].

The typical Western diet is characterized by a high n-6/n-3 ratio, which has been implicated in the development of obesity and related metabolic disorders [16]. Previous research has shown that reducing the n-6/n-3 ratio by increasing the consumption of n-3 fatty acids and/or reducing the intake of n-6 fatty acids can improve insulin sensitivity, decrease inflammation, and reduce the risk of developing NAFLD [39]. Our study provides further evidence to support the notion that a balanced n-6/n-3 ratio may be important for maintaining metabolic health. It is worth noting that the optimal n-6/n-3 ratio is still a matter of debate and may vary depending on individual characteristics such as genetics and lifestyle factors. Future research is needed to clarify the optimal n-6/n-3 ratio for preventing or treating metabolic disorders.

## 4. Materials and Methods

### 4.1. Diets

All three HF diets had identical contents of carbohydrates, protein, fiber, and micronutrients, and equal calories from macronutrients (20% derived from protein, 35% derived from carbohydrates, and 45% derived from fat). The fatty acid profile of each HF was analyzed by gas chromatography. The HF+n-6 and HF+n-3 diets had equal PUFA content (56% of total fat), and a similar amount of saturated and monounsaturated fatty acids (approximately 40% of total fat). The HF+n-6 diet contained 53.3% n-6 PUFAs primarily from soybean oil and safflower oil, while the HF+n-3 diet contained 43.8% n-3 PUFAs mainly from fish oil. These two HF+PUFA diets differed by the ratio of n-6/n-3 (HF+n-6, 20:1 vs. HF+n-3, 0.3:1). Chow diet (Labdiet, St. Louis, MO, USA) which contained 16% calories from fat was used as a low-fat control. The diet formula, fat composition, and fatty acid profile of the high-fat diets are listed in Supplementary Tables S1–S3, respectively.

### 4.2. Animals

Male 8-week-old C57BL/6J mice (Charles River Laboratory, Wilmington, MA, USA) were fed with a high-fat diet (HF, D12492, Research Diets Inc., New Brunswick, NJ, USA) or chow diet as low-fat control (C group). After 10 weeks, the C group continued feeding with the chow diet, and HF-fed mice were randomly distributed to 3 groups (*n* = 6–7) and fed with one of the following three diets for another 10 weeks: HF, HF+n-6, and HF+n-3. Detailed information on the experimental scheme is described in Figure 1A. Weekly records were maintained for body weight and food intake throughout the experiment. Upon completion of the study, mice underwent a 12 h fasting period before being euthanized to obtain liver tissues and blood samples. The Institutional Animal Care and Use Committee (IACUC) for Massachusetts General Hospital (MGH), represented by the Sub-committee on Research Animal Care (SRAC), thoroughly reviewed and approved all experimental procedures outlined in this study.

### 4.3. Body Composition

Body composition, encompassing lean tissue, fat, and body fluid, was assessed using the Bruker Minispec Live Mice (Bruker Optics Inc., Billerica, MA, USA). The methodology for this measurement has been detailed previously [40].

### 4.4. Glucose Tolerance Test (GTT) and Insulin Tolerance Test (ITT)

GTT and ITT were performed in the 8th week after the diet switch, and the procedures were described previously [41]. In summary, an intraperitoneal glucose tolerance test (GTT) involved the injection of 0.75 g of glucose per kg in mice following a 12 h fast. The insulin tolerance test (ITT) was performed continuously, with mice receiving an injection of 0.75 U of insulin per kg after a 6 h fasting period.

### 4.5. Semi-Quantitative PCR Analysis

Total mRNAs from cells/tissues were extracted with TRIzol^®^ (Invitrogen, Grand Island, NY, USA). The cDNA was synthesized by iScript^TM^ system (Bio-Rad, Hercules, CA, USA), and reverse transcription reaction was performed using a PTC-100 programmable thermal controller (MJ Research Inc., Waltham, MA, USA). A real-time PCR was performed using an iTaq^TM^ universal STBR^®^ Green Supermix (Bio-Rad, Hercules, CA, USA) in an Mx3005P qPCR thermocycler (Agilent Technologies, Santa Clara, CA, USA). All values were normalized by GAPDH expression and further analyzed using the ΔΔC_T_ method. The sequences of primers used in semi-quantitative PCR are listed in Supplementary Table S5.

### 4.6. Histology

The liver tissues were collected and fixed in 4% paraformaldehyde-PBS. The paraffin embedding and hematoxylin and eosin (H&E) staining were performed by pathology core in MGH. The slices were observed using an ECLIPSE E600 microscope (Micro Video Instruments Inc., Avon, MA, USA) at 100× and 400× magnification.

### 4.7. Hepatic Triglyceride Measurement

The liver was collected after 12 h fasting and triglyceride was measured following the manufacturer’s instruction (Triglyceride Colorimetric Assay Kit, Cayman, Ann Arbor, MI, USA).

### 4.8. Fatty Acid Profile

The liver or diet pellets were homogenized under liquid nitrogen and the lipid was extracted using 2:1 chloroform and methanol at 4 °C overnight. The weight of lipid content was measured after the samples were dried under nitrogen. The lipid fraction was further methylated by adding 1:1 of hexane and 14% boron trifluoride/methanol and heated at 100 °C for an hour. Fatty acid methyl esters were analyzed using an automated 6890N Network Gas Chromatograph equipped with a flame-ionization detector (Agilent Technologies, Palo Alto, CA, USA). Individual fatty acid was determined by retention time compared to a reference standard GLC461 (NuChek Prep., Elysian, MN, USA).

### 4.9. Statistical Analysis

Data are expressed as mean ± SEM for the number of replicates indicated. Statistical analysis was performed using one-way ANOVA followed by Fisher’s Least Significance Difference test, as appropriate (Prism 9 software, GraphPad, Boston, MA, USA). A significant difference was defined as *p* < 0.05.

## 5. Conclusions

Our study demonstrates the differential effects of n-6 PUFAs and n-3 PUFAs on body weight control for obesity, provides evidence for an interventional or therapeutic role for n-3 PUFAs in the management of obesity and associated fatty liver, and highlights the importance of a balanced n-6/n-3 PUFA ratio in the prevention and treatment of metabolic diseases. Thus, the information generated by our study may be valuable for the implementation of clinical interventions and the development of dietary guidelines to improve public health.

## Data Availability

Data is contained within the article and Supplementary Materials.

## Supplementary Materials

The following supporting information can be downloaded at: https://www.mdpi.com/article/10.3390/ijms242417261/s1.

## References

[1] Ratnayake W.M.N., Galli C. (2009). Fat and Fatty Acid Terminology, Methods of Analysis and Fat Digestion and Metabolism: A Background Review Paper. Ann. Nutr. Metab..

[2] Kang J.X. (2011). The omega-6/omega-3 fatty acid ratio in chronic diseases: Animal models and molecular aspects. World Rev. Nutr. Diet.

[3] Djuricic I., Calder P.C. (2021). Beneficial Outcomes of Omega-6 and Omega-3 Polyunsaturated Fatty Acids on Human Health: An Update for 2021. Nutrients.

[4] Kris-Etherton P.M., Krauss R.M. (2020). Public health guidelines should recommend reducing saturated fat consumption as much as possible: YES. Am. J. Clin. Nutr..

[5] Simopoulos A.P. (2006). Evolutionary aspects of diet, the omega-6/omega-3 ratio and genetic variation: Nutritional implications for chronic diseases. Biomed. Pharmacother..

[6] Costantini L., Molinari R., Farinon B., Merendino N. (2017). Impact of Omega-3 Fatty Acids on the Gut Microbiota. Int. J. Mol. Sci..

[7] Lepretti M., Martucciello S., Burgos Aceves M.A., Putti R., Lionetti L. (2018). Omega-3 Fatty Acids and Insulin Resistance: Focus on the Regulation of Mitochondria and Endoplasmic Reticulum Stress. Nutrients.

[8] Liput K.P., Lepczyński A., Ogłuszka M., Nawrocka A., Poławska E., Grzesiak A., Ślaska B., Pareek C.S., Czarnik U., Pierzchała M. (2021). Effects of Dietary n-3 and n-6 Polyunsaturated Fatty Acids in Inflammation and Cancerogenesis. Int. J. Mol. Sci..

[9] Saini R.K., Keum Y.S. (2018). Omega-3 and omega-6 polyunsaturated fatty acids: Dietary sources, metabolism, and significance—A review. Life Sci..

[10] Sinha S., Haque M., Lugova H., Kumar S. (2023). The Effect of Omega-3 Fatty Acids on Insulin Resistance. Life.

[11] Kang J.X., Weylandt K.H. (2008). Modulation of Inflammatory Cytokines by Omega-3 Fatty Acids. Subcell. Biochem..

[12] Albracht-Schulte K., Kalupahana N.S., Ramalingam L., Wang S., Rahman S.M., Robert-McComb J., Moustaid-Moussa N. (2018). Omega-3 fatty acids in obesity and metabolic syndrome: A mechanistic update. J. Nutr. Biochem..

[13] White P.J., Arita M., Taguchi R., Kang J.X., Marette A. (2010). Transgenic Restoration of Long-Chain n-3 Fatty Acids in Insulin Target Tissues Improves Resolution Capacity and Alleviates Obesity-Linked Inflammation and Insulin Resistance in High-Fat–Fed Mice. Diabetes.

[14] Bhattacharya A., Chandrasekar B., Rahman M., Banu J., Kang J.X., Fernandes G. (2006). Inhibition of inflammatory response in transgenic fat-1 mice on a calorie-restricted diet. Biochem. Biophys. Res. Commun..

[15] Mariamenatu A.H., Abdu E.M. (2021). Overconsumption of Omega-6 Polyunsaturated Fatty Acids (PUFAs) versus Deficiency of Omega-3 PUFAs in Modern-Day Diets: The Disturbing Factor for Their “Balanced Antagonistic Metabolic Functions” in the Human Body. J. Lipids.

[16] Simopoulos A.P. (2016). An Increase in the Omega-6/Omega-3 Fatty Acid Ratio Increases the Risk for Obesity. Nutrients.

[17] Kalupahana N.S., Claycombe K., Newman S.J., Stewart T., Siriwardhana N., Matthan N., Lichtenstein A.H., Moustaid-Moussa N. (2010). Eicosapentaenoic acid prevents and reverses insulin resistance in high-fat diet-induced obese mice via modulation of adipose tissue inflammation. J. Nutr..

[18] Huang X.F., Xin X., McLennan P., Storlien L. (2004). Role of fat amount and type in ameliorating diet-induced obesity: Insights at the level of hypothalamic arcuate nucleus leptin receptor, neuropeptide Y and pro-opiomelanocortin mRNA expression. Diabetes Obes. Metab..

[19] Softic S., Cohen D.E., Kahn C.R. (2016). Role of Dietary Fructose and Hepatic De Novo Lipogenesis in Fatty Liver Disease. Dig. Dis. Sci..

[20] Caballero B. (2007). The Global Epidemic of Obesity: An Overview. Epidemiol. Rev..

[21] World Health Organization Obesity and Overweight. http://www.who.int/news-room/fact-sheets/detail/obesity-and-overweight.

[22] Kopelman P.G. (2000). Obesity as a medical problem. Nature.

[23] Bray G.A., Kim K.K., Wilding J.P.H., World Obesity Federation (2017). Obesity: A chronic relapsing progressive disease process. A position statement of the World Obesity Federation. Obes. Rev..

[24] Buzzetti E., Pinzani M., Tsochatzis E.A. (2016). The multiple-hit pathogenesis of non-alcoholic fatty liver disease (NAFLD). Metabolism.

[25] Younossi Z.M., Blissett D., Blissett R., Henry L., Stepanova M., Younossi Y., Racila A., Hunt S., Beckerman R. (2016). The economic and clinical burden of nonalcoholic fatty liver disease in the United States and Europe. Hepatology.

[26] Africa J.A., Newton K.P., Schwimmer J.B. (2016). Lifestyle Interventions Including Nutrition, Exercise, and Supplements for Nonalcoholic Fatty Liver Disease in Children. Dig. Dis. Sci..

[27] Rebello C.J., Greenway F.L. (2019). Obesity medications in development. Expert Opin. Investig. Drugs.

[28] He X.-X., Wu X.-L., Chen R.-P., Chen C., Liu X.-G., Wu B.-J., Huang Z.-M. (2016). Effectiveness of Omega-3 Polyunsaturated Fatty Acids in Non-Alcoholic Fatty Liver Disease: A Meta-Analysis of Randomized Controlled Trials. PLoS ONE.

[29] Shahidi F., Ambigaipalan P. (2018). Omega-3 Polyunsaturated Fatty Acids and Their Health Benefits. Annu. Rev. Food Sci. Technol..

[30] Innes J.K., Calder P.C. (2020). Marine Omega-3 (N-3) Fatty Acids for Cardiovascular Health: An Update for 2020. Int. J. Mol. Sci..

[31] LeMieux M.J., Kalupahana N.S., Scoggin S., Moustaid-Moussa N. (2015). Eicosapentaenoic Acid Reduces Adipocyte Hypertrophy and Inflammation in Diet-Induced Obese Mice in an Adiposity-Independent Manner. J. Nutr..

[32] Alexander-Aguilera A., Berruezo S., Hernández-Diaz G., Angulo O., Oliart-Ros R. (2011). Dietary n-3 polyunsaturated fatty acids modify fatty acid composition in hepatic and abdominal adipose tissue of sucrose-induced obese rats. J. Physiol. Biochem..

[33] Belzung F., Raclot T., Groscolas R. (1993). Fish oil n-3 fatty acids selectively limit the hypertrophy of abdominal fat depots in growing rats fed high-fat diets. Am. J. Physiol. Integr. Comp. Physiol..

[34] Sato A., Kawano H., Notsu T., Ohta M., Nakakuki M., Mizuguchi K., Itoh M., Suganami T., Ogawa Y. (2010). Antiobesity Effect of Eicosapentaenoic Acid in High-Fat/High-Sucrose Diet–Induced Obesity. Diabetes.

[35] Capanni M., Calella F., Biagini M.R., Genise S., Raimondi L., Bedogni G., Svegliati-Baroni G., Sofi F., Milani S., Abbate R. (2006). Prolonged n-3 polyunsaturated fatty acid supplementation ameliorates hepatic steatosis in patients with non-alcoholic fatty liver disease: A pilot study. Aliment. Pharmacol. Ther..

[36] Sofi F., Giangrandi I., Cesari F., Corsani I., Abbate R., Gensini G.F., Casini A. (2010). Effects of a 1-year dietary intervention with n-3 polyunsaturated fatty acid-enriched olive oil on non-alcoholic fatty liver disease patients: A preliminary study. Int. J. Food Sci. Nutr..

[37] Spadaro L., Magliocco O., Spampinato D., Piro S., Oliveri C., Alagona C., Papa G., Rabuazzo A., Purrello F. (2008). Effects of n-3 polyunsaturated fatty acids in subjects with nonalcoholic fatty liver disease. Dig. Liver Dis..

[38] Guo X.-F., Wang C., Yang T., Ma W.-J., Zhai J., Zhao T., Xu T.-C., Li J., Liu H., Sinclair A.J. (2022). The effects of fish oil plus vitamin D3 intervention on non-alcoholic fatty liver disease: A randomized controlled trial. Eur. J. Nutr..

[39] Masterton G.S., Plevris J.N., Hayes P.C. (2010). Review article: Omega-3 fatty acids—A promising novel therapy for non-alcoholic fatty liver disease. Aliment. Pharmacol. Ther..

[40] Taicher G.Z., Tinsley F.C., Reiderman A., Heiman M.L. (2003). Quantitative magnetic resonance (QMR) method for bone and whole-body-composition analysis. Anal. Bioanal. Chem..

[41] Ayala J.E., Samuel V.T., Morton G.J., Obici S., Croniger C.M., Shulman G.I., Wasserman D.H., McGuinness O.P. (2010). Standard operating procedures for describing and performing metabolic tests of glucose homeostasis in mice. Dis. Model. Mech..

