# Supplementary material for: Differential Interventional Effects of Omega-6 and Omega-3 Polyunsaturated Fatty Acids on High Fat Diet-Induced Obesity and Hepatic Pathology
Source: Int J Mol Sci. 2023 Dec 8;24(24):17261. doi: 10.3390/ijms242417261 (PMC10743920; doi:10.3390/ijms242417261)
Supplement: Supplementary file 1 [file ijms-24-17261-s001.zip › ijms-2717019-supplementary.pdf]

Supplementary Table S1. Diet formula

Diet	HF		HF+n-6		HF+n-3	
	**gm%**	**kcal%**	**gm%**	**kcal%**	**gm%**	**kcal%**
Protein	24	20	24	20	24	20
Carbohydrate	41	35	41	35	41	35
Fat	24	45	24	45	24	45
**Total**		100		100		100
**kcal/gm**	**4.73**		**4.73**		**4.73**	
**Ingredient**	**g**	**kcal**	**g**	**kcal**	**g**	**kcal**
Casein	233.1	932	233.1	932	233.1	932
L-Cystine	3.5	14	3.5	14	3.5	14
		0		0		0
Corn Starch	84.8	339	84.8	339	84.8	339
Maltodextrin	116.5	466	116.5	466	116.5	466
Sucrose	201.4	806	201.4	806	201.4	806
Cellulose	58.3	0	58.3	0	58.3	0
Soybean Oil	29.1	262	87.3	786	9.4	85
Safflower Oil	0.0	0	96.7	870	0.0	0
Fish Oil	0.0	0	0.0	0	129.1	1162
Lard	206.9	1862	52.0	468	97.5	877
Mineral Mix	11.7	0	11.7	0	11.7	0
DiCalcium Phosphate	15.1	0	15.1	0	15.1	0
Calcium Carbonate	6.4	0	6.4	0	6.4	0
Potassium Citrate, 1 H_2O	19.2	0	19.2	0	19.2	0
Vitamin Mix	11.7	47	11.7	47	11.7	47
Choline Bitartrate	2.3	0	2.3	0	2.3	0
**Total**	1000.0	4728	1000.0	4728	1000.0	4728

Supplementary Table S2. Fat composition

	HF	HF+n-6	HF+n-3
**Component (g%)**			
Soybean Oil	3.2	12.7	4
Safflower Oil	0	16.4	0
Fish Oil	0	0	13.3
Lard	31.6	5.8	17.5
Total Fat	34.8	34.9	34.8
**Fatty Acid Composition (% fat)**			
Saturated	42.3	20.1	19.8
Monounsaturated	37	21.8	18.7
n-6 PUFA	16.2	53.3	12.2
n-3 PUFA	1.6	2.7	43.8
Total PUFA	17.8	56	56
n-6/n-3 ratio	10:1	20:1	0.3:1

Supplementary Table S3. Fatty acid composition in diets

Fatty Acids	HF	HF+n-6	HF+n-3
C4:0	0.018	0.055	0.006
C8:0	0.110	0.121	0.047
C10:0	0.141	0.145	0.061
C12:0	1.370	0.546	0.638
C14:0	0.041	0.124	0.014
C16:0	22.790	12.430	10.892
C18:0	15.031	6.403	7.36
C17:0	0.399	0.258	0.180
C17:1	0.042	0.125	0.014
C16:1	1.745	0.590	1.091
C18:1	32.786	20.978	16.883
C18:2 (n-6)	21.280	53.210	10.400
C18:3 (n-6)	0.041	0.133	0.180
C20:2 (n-6)	ND	ND	ND
C20:3 (n-6)	ND	ND	ND
C20:4 (n-6)	ND	ND	1.560
C22:2 (n-6)	ND	ND	ND
C18:3 (n-3)	0.889	2.666	0.682
C20:3 (n-3)	ND	ND	ND
C20:5 (n-3)	ND	ND	31.869
C22:5 (n-3)	ND	ND	1.805
C22:6 (n-3)	ND	ND	9.468

ND means the fatty acid was not detected in the diet.

Supplementary Table S4. Liver fatty acid profile

Fatty Acids	C	HF	HF+n-6	HF+n-3
**C12:0**	0.293 ± 0.04[ab]	0.338 ± 0.03[a]	0.274 ± 0.02[ab]	0.209 ± 0.02[b]
**C16:0**	27.448 ± 0.26[a]	25.326 ± 0.62[b]	21.351 ± 0.68[c]	23.818 ± 0.35[b]
**C17:0**	0.351 ± 0.03[a]	0.264 ± 0.02[b]	0.196 ± 0.01[c]	0.251 ± 0.01[bc]
**C18:0**	11.12 ± 0.50[b]	10.180 ± 1.16[b]	8.629 ± 0.83[b]	13.741 ± 0.57[a]
**C20:0**	0.390 ± 0.02[a]	0.364 ± 0.03[a]	0.263 ± 0.02[b]	0.367 ± 0.02[a]
**C22:0**	0.369 ± 0.04[b]	0.297 ± 0.05[bc]	0.166 ± 0.03[c]	0.569 ± 0.06[a]
**C24:0**	0.170 ± 0.01[a]	0.074 ± 0.01[b]	0.058 ± 0.01[b]	0.143 ± 0.01[a]
**C14:1**	0.132 ± 0.01[a]	0.104 ± 0.00[b]	0.086 ± 0.01[c]	0.101 ± 0.00[bc]
**C16:1**	2.714 ± 0.48[a]	1.561 ± 0.26[b]	0.850 ± 0.12[bc]	0.432 ± 0.05[c]
**C18:1**	13.849 ± 1.20[bc]	24.486 ± 2.38[a]	14.998 ± 1.36[b]	9.318 ± 0.69[c]
**C20:1**	0.407 ± 0.01[b]	0.512 ± 0.05[a]	0.398 ± 0.03[b]	0.215 ± 0.01[c]
**C22:1**	0.090 ± 0.01[a]	0.066 ± 0.00[b]	0.037 ± 0.00[c]	ND
**C24:1**	0.210 ± 0.03[a]	0.094 ± 0.02[b]	0.070 ± 0.01[b]	0.194 ± 0.02[a]
**C18:2 (n-6)**	16.686 ± 0.57[b]	16.703 ± 0.45[b]	34.090 ± 0.53[a]	11.853 ± 0.30[c]
**C18:3 (n-6)**	0.272 ± 0.02[c]	0.413 ± 0.03[b]	0.696 ± 0.04[a]	0.067 ± 0.01[d]
**C20:2 (n-6)**	0.300 ± 0.03[b]	0.312 ± 0.01[b]	0.525 ± 0.02[a]	0.140 ± 0.01[c]
**C20:3 (n-6)**	1.547 ± 0.06[a]	1.047 ± 0.04[b]	1.605 ± 0.05[a]	0.293 ± 0.02[c]
**C20:4 (n-6)**	10.595 ± 0.63[a]	11.294 ± 1.12[a]	9.509 ± 0.80[a]	6.240 ± 0.27[b]
**C18:3 (n-3)**	0.414 ± 0.03[b]	0.351 ± 0.02[b]	0.820 ± 0.04[a]	0.364 ± 0.05[b]
**C20:3 (n-3)**	ND	ND	0.033 ± 0.00[a]	0.029 ± 0.00[a]
**C20:5 (n-3)**	1.325 ± 0.06[b]	0.213 ± 0.02[c]	0.226 ± 0.02[c]	6.571 ± 0.24[a]
**C22:5 (n-3)**	0.829 ± 0.09[b]	0.414 ± 0.02[c]	0.435 ± 0.03[c]	1.751 ± 0.08[a]
**C22:6 (n-3)**	10.491 ± 0.32[b]	5.584 ± 0.56[c]	4.686 ± 0.40[c]	23.332 ± 0.43[a]
**SFA**	40.140 ± 0.48[a]	36.843 ± 0.71[b]	30.937 ± 0.40[c]	39.098 ± 0.49[a]
**MUFA**	17.402 ± 1.65[b]	26.823 ± 2.66[a]	16.439 ± 1.49[b]	10.261 ± 0.72[c]

**PUFA**	42.458 ± 1.25[b]	36.333 ± 1.97[c]	52.624 ± 1.30[a]	50.640 ± 0.32[a]
**n-6 PUFA**	29.127 ± 1.12[b]	29.357 ± 1.47[b]	45.729 ± 0.91[a]	18.526 ± 0.25[c]
**n-3 PUFA**	13.059 ± 0.25[b]	6.563 ± 0.54[c]	6.199 ± 0.41[c]	32.047 ± 0.43[a]
**n-6/n-3**	2.232 ± 0.08[c]	4.553 ± 0.18[b]	7.535 ± 0.41[a]	0.579 ± 0.01[d]

Values represent mean ± S.E.M. Different letters indicate significant difference.

ND means the fatty acid was not detected in the sample.

Supplementary Table S5. Primer sequences

Gene Symbol	Gene Name	F, Forward primer<br>R, Reverse primer
Gapdh	Glyceraldehyde 3-phosphate dehydrogenase	F: 5'- CAACTCCCACTCTTCCACCT -3'<br>R: 5'- GAGTTGGGATAGGGCCTCTC -3'
Acc	Acetyl-CoA carboxylase	F: 5'- GGGGAGAAAACAGGGAGGAA -3'<br>R: 5'- CCTCAGGCTCACATCTGCTA-3'
Fas	Fatty acid synthase	F: 5'- CCCAATGGTTGCTGATTACAAAT -3'<br>R: 5'- CTACTTTGATCG CACTTTGGTATTCT -3'
Srebp1c	Sterol regulatory element binding transcription factor 1	F: 5'- CCCACCTCAAACCTGGATCT-3’<br>R: 5'- AAGCAGCAAGATGTCCTCCT-3'
Scd-1	Stearoyl-CoA desaturase 1	F: 5'- CGGGGACAGATATGGTGTGA -3’<br>R: 5'- GCCCCTCAACCTCACTAGTT -3'
